# How much biotic nativeness matters across human demographic groups

**DOI:** 10.1111/cobi.70197

**Published:** 2025-12-27

**Authors:** Harold N. Eyster, Rachelle K. Gould

**Affiliations:** ^1^ Gund Institute for Environment University of Vermont Burlington Vermont USA; ^2^ Department of Plant Biology University of Vermont Burlington Vermont USA; ^3^ The Nature Conservancy Boulder Colorado USA; ^4^ Rubenstein School of Environment and Natural Resources University of Vermont Burlington Vermont USA

**Keywords:** bird declines, community engagement, environmental justice, House Sparrow and European Starling, inclusion, non‐native biodiversity, public perception, species management, conservación, declinación de aves, diversidad, equidad, equidad e inclusión no nativa, marginalización, 非本土物种, 边缘化, 公平性, 保护, 多样性、公平性与包容性, 鸟类数量下降

## Abstract

Many central concepts of conservation biology—such as nativeness—are structured by ecological and social factors. However, the social consequences of using these concepts to make conservation decisions remain inadequately understood. Some researchers argue that nativeness, rather than acting as an objective proxy for important ecological relationships, may instead mask social and cultural values about which species belong in a given ecosystem. Yet, even as many non‐native species decline, experts often prioritize the conservation of native species. We assessed the perceptions of people (*n* = 600) in Metro Vancouver, Canada, regarding local declines of native and non‐native birds. We measured ecological grief (feelings of loss associated with ecological changes) and loss of cultural ecosystem service (nonmaterial benefits people derive from relationships with nature) associated with documented declines in 2 native and 2 non‐native birds. We measured variations in perceptions across differences in nature experiences and sociodemographics. We used a 2‐treatment experimental design in which we informed only half the participants about species’ nativeness. Perceptions of loss differed among respondents based on their familiarity with birds, experiences with birds, and the native status of the bird. However, the effect of nativeness on feelings of loss was not moderated by ecological knowledge, whether a respondent was an urbanite, or experiences with birds. Instead, race was the strongest moderator of the effect of nativeness on feelings of loss. Only White people reported greater grief for declines in native species than non‐native species, even when accounting for education, income, and other variables. Although native status may often be a useful heuristic for inference, relying on it for conservation decision‐making may have unintended sociodemographic and equity consequences. Our results also demonstrate how pairing ecological grief and cultural ecosystem service questions with documented ecological declines can elucidate human–nature relationships, such as those between people and non‐native birds.

## Abstract (es)

Importancia de cuán nativa es una especie para los grupos demográficos humanos

**Resumen:**

Muchos conceptos centrales de la biología de la conservación, como cuán nativa es una especie, se estructuran en función de factores ecológicos y sociales. Sin embargo, no comprendemos adecuadamente las consecuencias sociales de utilizar estos conceptos para tomar decisiones en materia de conservación. Algunos investigadores sostienen que cuán nativa es una especie, en lugar de ser un indicador objetivo de relaciones ecológicas importantes, puede enmascarar valores sociales y culturales sobre qué especies pertenecen a un ecosistema determinado. Sin embargo, incluso cuando muchas especies no nativas están en declive, los expertos suelen dar prioridad a la conservación de las especies nativas. Evaluamos las percepciones de las personas (n = 600) en el área metropolitana de Vancouver, Canadá, sobre la disminución local de aves nativas y no nativas. Medimos el dolor ecológico (sentimientos de pérdida asociados con los cambios ecológicos) y la pérdida de servicios ambientales culturales (beneficios no materiales que las personas obtienen de la naturaleza) asociados con la disminución documentada de dos especies de aves nativas y dos no nativas. Medimos las variaciones en las percepciones a través de las diferencias en las experiencias con la naturaleza y los datos sociodemográficos. Utilizamos un diseño experimental de dos tratamientos en el que sólo informamos a la mitad de los participantes sobre el origen nativo de las especies. Las percepciones de pérdida diferían entre los encuestados en función de su familiaridad con las aves, sus experiencias con ellas y cuán nativas eran. Sin embargo, el efecto de esto en los sentimientos de pérdida no se vio moderado por los conocimientos ecológicos, el hecho de que el encuestado fuera urbanita o sus experiencias con las aves. En cambio, la raza fue el moderador más fuerte del efecto de cuán nativa es la especie sobre los sentimientos de pérdida. Solo las personas blancas manifestaron un mayor dolor por la disminución de las especies nativas que por las especies no nativas, incluso teniendo en cuenta la educación, los ingresos y otras variables. Aunque el estatus de autenticidad puede ser a menudo una heurística útil para la inferencia, basarse en él para la toma de decisiones sobre conservación puede tener consecuencias sociales y de equidad no deseadas. Nuestros resultados también demuestran cómo la combinación de cuestiones ecológicas y culturales sobre los servicios ambientales con la disminución ecológica documentada puede esclarecer las relaciones entre el ser humano y la naturaleza, como las que existen entre las personas y las aves no nativas.

## 物种本土性在不同人群中的重要性

**【摘要】** 保护生物学的许多核心概念, 如本土性, 是由生态和社会因素共同塑造的。然而, 运用这些概念来制定保护决策的社会后果尚未得到充分认识。部分研究者认为, 本土性并非重要生态关系的客观替代指标, 反而可能掩盖特定物种在生态系统中的归属所涉及的社会文化价值。然而, 即便许多非本土物种数量已经下降, 专家们仍通常优先保护本土物种。本研究评估了加拿大温哥华都会区600名居民对本土及非本土鸟类数量下降的认知, 通过测量生态悲伤情绪(由生态变化引发的失落感)与文化生态系统服务(人类从自然中获得的非物质效益)丧失, 评估了两种本土鸟类和两种非本土鸟类已有记录的数量下降所引发的影响。我们分析了存在不同自然体验和社会人口特征的受访者之间的认知差异。研究采用双处理实验设计, 仅告知了半数受访者物种的本土性状态。受访者的丧失感受因其对鸟类的熟悉程度、与鸟类有关的经历及鸟类本土性状态而异。然而, 物种本土性对人们丧失感受的影响不受生态知识水平、受访者是否为城市居民, 或是与鸟类有关经历的调节。相反, 种族才是人们因物种本土性状态影响丧失感受的最强调节因素。只有白人群体对本土物种丧失的悲伤情绪显著强于非本土物种, 即便在控制教育水平、收入及其他变量后依然如此。尽管物种本土性常被用作启发式推断依据, 但将其作为保护决策依据可能会引发意想不到的社会公平后果。我们的研究还表明, 将生态悲伤情绪及文化生态系统服务问题与已记录的生态退化现象相结合, 有助于阐释人与自然的关系, 例如人类与非本土鸟类之间的关系。**【翻译:胡怡思;审校:聂永刚】**

## INTRODUCTION

Conservation priorities are often guided by scientific experts’ understandings of how to promote biodiversity (Turnhout et al., 2019). Yet, what species count towards biodiversity is not always straightforward because biodiversity and many other concepts central to conservation biology can only be defined by considering both social and ecological factors (Castelló & Santiago‐Ávila, 2023; Eyster et al., 2023). As conservation science becomes increasingly interdisciplinary, it is now possible to investigate the social and equity consequences of how conservation experts define and use these socially structured concepts (Vercammen et al., 2025).

The concept of biotic nativeness provides a notable example of a socially structured concept in conservation (Chew & Hamilton, 2011). How nativeness is defined and used may have particularly large socioecological effects because it determines the conservation (or lack thereof) of the many so‐called non‐native species that are declining rapidly (Berigen et al., 2021; Rosenberg et al., 2019). Biologists typically define non‐native species as species that humans transported to a new location (Chew & Hamilton, 2011) and often use nativeness as an indicator of conservation value and, ultimately, of which species belong. For example, biodiversity metrics typically exclude non‐native species (Berthon et al., 2021; Pyšek & Richardson, 2010; Richardson, 2011), including in legal conservation structures (e.g., International Migratory Bird Treaty Act in the USA) and community science tools (e.g., eBird, 2023).

Yet, nativeness can be an ineffective indicator of conservation value. Western Cattle‐Egrets (*Ardea* *ibis*) and House Sparrows (*Passer domesticus*) illustrate how native and non‐native species do not necessarily have distinct ecological roles. Without any direct assistance from humans, Western Cattle‐Egrets likely arrived in Florida from South America around 1941 on their own wings (Crosby, 1972; Daniel et al., 2022). Nearly a century earlier, in 1851, people released 16 House Sparrows from England in New York with hopes that they would eat urban insect pests and serve as reminders of home for European human immigrants (Broadhead, 1971; Moulton et al., 2010). Both species have since spread across North America (Daniel et al., 2022; Moulton et al., 2010).

Although both species have been in North America for less time than most of the continent's other avifauna, the circumstances of the arrival of each species mean that the egret is considered native and is protected under the US Migratory Bird Treaty Act 1918, whereas the sparrow is not (16 USC Chapter 7, Subchapter II) (eBird, 2023). This difference exists even though both have relied on human habitat modifications to thrive (Daniel et al., 2022; Moulton et al., 2010). For example, cattle and tractors, which stir up invertebrates, increased foraging efficiency for egrets and thus enabled their range expansion (Daniel et al., 2022; Mukherjee, 2000). Similarly, House Sparrows relied on horse manure and spilled grain to survive and expand their range in the 1800s (Moulton et al., 2010). These examples, among many others, show that ecosystems are neither static nor natural and are directly and indirectly co‐constructed by humans (Eyster et al., 2023; Lorimer, 2015; Marris, 2013; Watts, 2013).

The spread of both Western Cattle‐Egrets and House Sparrows may negatively affect so‐called native birds (e.g., yellow‐rumped warblers [*Setophaga coronata*] and eastern bluebirds [*Sialia sialis*], respectively) (Cunningham, 1965; Radunzel et al., 1997). But ecological relationships are immensely complex. Native birds also have detrimental effects on other native birds. For example, a study of nest success of eastern bluebirds showed that House Sparrows cause many nest failures, but the native Northern House Wren (*Troglodytes aedon*) causes the most nest failures (Radunzel et al., 1997). Moreover, non‐native species have a range of desirable impacts; they can “contribute to achieving conservation goals” by, for example, catalyzing ecosystem restoration (Schlaepfer et al., 2011, p. 430); create resiliency in the face of climate change (Eyster & Wolkovich, 2021); help form a novel and flourishing Anthropocene biota (Thomas, 2020); and form strong relationships with people, such as in the case of monk parakeets in England (*Myiopsitta monachus*) (Crowley et al., 2019). A recent review concluded that the “…benefits of non‐native species are diverse, frequent, and often of large magnitude” (Sax et al., 2022, p. 1058). Yet, conservation bodies continue to denounce non‐native species, including in the United States (Migratory Bird Treaty Act of 1918), Australia (*Environment Protection and Biodiversity Conservation Act 1999* [https://www.legislation.gov.au/C2004A00485/latest/versions]), the United Kingdom (NNSS, 2025), and China (National People's Congress of the People's Republic of China, 2020). If nativeness does not reflect fundamental ecological relationships, what does it represent and how can conservationists decide whether it is an important category for addressing the biodiversity crisis?

Today's native versus non‐native distinctions were codified by 19th‐century White, male British botanists (Chew & Hamilton, 2011; Watson, 1835, 1847). Historical analyses suggest that the originators of the nativeness concept aimed to create or reinforce a boundary between the natural and human in taxonomic lists, and they included specific analogs to human nationalities and racist ideas that reflected English common law on human citizenship and rights (Chew & Hamilton, 2011; see Biermann & Mansfield [2014] for a more general critique). The term came to mean that a species was not known to have been directly assisted by humans to arrive at a location any time in its dispersal history. Asterisks in species lists “denoted suspicion of human dispersal…” (see examples in Chew & Hamilton [2011, p. 37]). This practice continues today, with asterisks used to denote non‐native species in modern taxonomic checklists (e.g., eBird, 2023). As the egret and sparrow example show, today's concept of nativeness does not reflect fundamental ecological relationships; instead, it reflects powerful 19th‐century colonial and imperialist ideas about belonging, race, and human migration. The nativeness concept hearkens back to a view that species are immutable, people and nature are separate, and ranges are static unless disrupted by humans (Chew & Hamilton, 2011; Thomas, 2020). Consequently, biodiversity is replete with values and advances a particular vision for how landscapes should look—usually visions held by those who have power (where power is “social relation built on the asymmetrical distribution of resources and risks” [Paulson et al., 2003, p. 205; Biermann & Mansfield, 2014; Eyster, González, et al., 2024; Munro et al., 2019; Pascual et al., 2021; Taylor, 2016; Wallach et al., 2018]).

Biodiversity is a socially and politically structured concept that is understood, measured, and valued differently across geographies, communities, and demographics (Wardell‐Johnson, 2011). People have diverse relationships with nature and wildlife—variously positive or negative and involving different salient species and landscapes (Horne et al., 2005; Ley, 1995; Trigger & Head, 2010; Wardell‐Johnson, 2011)—and these relationships often covary with dimensions and structures of privilege and political, economic, and social power, such as recognized expertise, indigeneity, gender, education, wealth, race, and class (Biermann & Mansfield, 2014; Taylor, 2016). If relationships with non‐native species also vary across people in ways that align with power differentials, then when conservationists prioritize the nativeness category they may overlook declines in non‐native species and implicitly and unintentionally foster landscapes that represent the visions and ideas of those in power, a than local residents (Yaka, 2019). Yet, invasion biologists have long denounced the idea that xenophobia shapes ideas about so‐called non‐native species (Simberloff, 2003; cf. Larson, 2005), and they only rarely consider the relationships that marginalized people have with non‐native species (Kapitza et al., 2019; Subramaniam, 2001).

We investigated whether use of the nativeness concept to guide conservation of birds might lead to landscapes that are not aligned with the values and experiences of local residents, especially those who have been marginalized, by assessing people's perceptions (*n* = 600) of local declines in native and non‐native birds and what factors are associated with these perceptions. Table 1 provides background on the social science frameworks we used. In sum, we measured the association of ecological grief (feelings of loss associated with certain ecological changes) and loss of cultural ecosystem service (nonmaterial benefits that humans derive from relationships with nature) with documented, colocated population declines in 2 native and 2 non‐native bird species in Metro Vancouver, British Columbia, Canada (approximately 3 million people). Studies that present participants with measured place‐based ecological data and assess associated values are rare, but, we suggest, they can provide important theoretical insight to guide ecological management. We investigated how these perceptions varied across differences in personal experiences with nature and sociodemographic factors. We used a 2‐treatment experimental design in which we informed half the participants about species’ nativeness and collected knowledge about nativeness from the other half.

**TABLE 1:** Social science frameworks used to measure and make salient people's ecologically specific relationships with nature. Approaches respond to the fact that many studies of the nuances of peoples’ relationships with nature are quite disconnected from studies of the ecological nuances of places (Chan & Satterfield, 2020; Gould et al., 2020; Kluger et al., 2020), which decreases their usefulness for ecosystem management (IPBES, 2022). The tools listed here can help illuminate ecological nuance in multiple ways by drawing on real ecological change and associated feelings of loss.

Concept or tool	Definition	Advantages	How ecological specificity can be incorporated	How it can make human–nature relationships salient
Ecological change scenarios	Ecological changes presented to survey or interview participants	Help bridge the gap between social and ecological research	Often includes ecological specificity (e.g., images of different ecological contexts [Graves et al., 2017]), projected landcover change (Grêt‐Regamey et al., 2007), or action–choice situations (Gould et al. 2014)	Although scenarios are often hypothetical, they can incorporate real, measured ecological change, thereby making the scenarios appear more real and the responses better‐suited to informing actual ecological management decisions
Ecological grief	Feelings of loss associated with certain ecological changes	Can target feelings triggered by loss of meaningful interdependent relationships (Cunsolo, 2017a, 2017b; Cunsolo & Ellis, 2018)	Prompts for grief (i.e., the relational object or source of inquired‐after grief) can be ecologically specific	Grief framing encourages consideration of lost connections, which can make taken‐for‐granted relationships salient (Gould & Schultz, 2021)
Cultural ecosystem services	“[E]cosystems’ contributions to the non‐material benefits… that arise from human–ecosystem relationships” (Chan et al., 2012, p. 9)	Provide robust research tradition and large bank of tested questions for characterizing nonmaterial human–nature relationships (McElwee et al., 2025)	Research can inquire about cultural ecosystem services associated with specific species, ecosystems, or ecosystem types or conditions	Ecological change can be framed as loss of cultural ecosystem services to make nonmaterial human–nature relationships (e.g., with neighborhood birds) salient and measurable

### Positionality

Research is a social practice that is influenced by researcher experiences and social location and identity relative to the study subjects, that is, by researcher positionalities (Boyce et al., 2021; Latour & Woolgar, 1979; Montana et al., 2020; Phurisamban et al., 2025). H.N.E. spent 5 years living as a White settler in the City of Vancouver and conducting social and ecological research, including bird surveys, across the region. The stark declines in non‐native birds H.N.E. documented (Eyster, Chan, et al., 2024) and the many conversations H.N.E. had with residents about their relationships with birds and the differences between how conservationists, birders, and local residents spoke about non‐native species contributed to the design of the study. Both of us have interdisciplinary training and commitments to anticolonial research that inspired us to seek out and interrogate the different ways critical social scientists and ecologists discuss the concept of biological nativeness.

## METHODS

### Relational approach

To develop our questionnaire and analyze responses to it, we adopted a relational methodology. Following ecological psychology and other relational scholarship, we treated each participant's responses to the ecologically specific ecological grief and cultural ecosystem service questions as reflections of specific relationships among each participant, the wildlife and ideas evoked by the survey, and salient social and environmental factors (Eyster et al., 2023; Heft, 2013; Winkel et al., 2009). We recognized the relationship specificity of each ecological grief and cultural ecosystem service grief item (Gibson, 1979; Lewin, 1939) by treating each item as an independent random effect in our models (both intercepts and slopes). Our approach is consistent with calls in the nature connectedness literature to treat connection to nature as multidimensional (Tam, 2013). Thus, although we did adapt items from scales that had been assessed for reliability and validity (see below), we did not assess reliability and validity across items because we did not adopt the assumption that constructs are consistently represented by items. This contrasts with much psychology research, which treats items as representing dimensions of generalized constructs and so measures reliability across dimensions and then combines dimensions to form a generalized construct (e.g., Dietz et al., 2005; Dunlap, 2008).

### Analysis plan and hypotheses

Preregistration (i.e., an analysis plan documented before data collection) is considered best practice in psychology and ensures that researchers do not inadvertently identify false positives (Simmons et al., 2021). However, exploratory analyses is also an important part of science. Indeed, iterative, exploratory analyses often produce the most robust statistical inference (Box, 1997; Gelman & Loken, 2013; Tukey, 1980). The combination of preregistered (deductive) and exploratory (inductive) research may improve inference (e.g., Hatta et al., 2018). In this study, we first conducted a preregistered analysis, but this model did not fully explain the variation in perceptions of nativeness because the relationships that we hypothesized to be important were not, so we carried out follow‐up exploratory analyses.

### Study population and recruitment

We surveyed residents of Metro Vancouver, British Columbia, Canada, with an online questionnaire on Qualtrics in July–August 2023. We chose this location and time to correspond with the temporal and spatial extent of a study of Metro Vancouver's bird declines conducted from 1997 to 2020 (Eyster, Chan, et al., 2024). As in the rest of North America, many of Vancouver's non‐native bird species have declined significantly (Eyster, Chan, et al., 2024). Yet, neither local municipal (e.g., City of Vancouver, 2020) nor national regulations foster the conservation of non‐native species (Government of Canada, 2019). Metro Vancouver sits on the traditional, ancestral, and unceded lands of 11 First Nations, including the Hwlitsum, q̓ic̓əy̓(Katzie), q̓ʷɑ:n̓ƛ̓ən̓ (Kwantlen), kʷikʷəƛ̓əm (Kwikwetlem), máthxwi (Matsqui), xʷməθkʷəy̓əm (Musqueam), qiqéyt (Qayqayt), Semiahmoo, Sḵwx̱wú7mesh Úxwumixw (Squamish), scəw̓​aθən məsteyəxʷ (Tsawwassen), and səlilwətaɬ​ (Tsleil‐Waututh). These people have stewarded this land since time immemorial.

Study participants were members of an online panel recruited through various avenues by Leger Opinion (Montreal, QC). Although online panels can be associated with low data quality, proper quality checks can provide samples that meet standards for psychological studies (Buhrmester et al., 2011). (Our survey was carried out in summer 2023, before the widespread use of generative artificial intelligence.) A total of 722 participants began our survey. We excluded 19 respondents who did not live in Metro Vancouver. To ensure high‐quality data, we excluded 97 participants who incorrectly answered the attention check question (“Please select strongly agree.”) and 6 participants who did not complete the survey. Our final survey sample size was 600. We targeted 600 participants because the survey company was confident this sample size was achievable in Metro Vancouver. Because excluded participants did not answer demographic questions, we do not know how our initial and final samples differed. The survey was approved by the University of Vermont Institutional Review Board (STUDY00002618). The full survey is in Appendix S3.

### Survey design

The questionnaire had 3 main sections. The first section addressed locally estimated declines in each of 4 declining bird species, 2 native (American Robin [*Turdus migratorius*, L.] and Barn Swallow [*Hirundo rustica*, L.]) and 2 non‐native species (House Sparrow and European Starling [*Sturnus vulgaris*, L.]). The population trend estimates for each species were based on data collected from the same region in which the survey participants resided (Metro Vancouver) (Eyster, Chan, et al., 2024). European starlings were intentionally released in New York in the 1890s as part of a plan to bring species in William Shakespear's plays to North America and, by the 1960s, were prevalent in British Columbia (Johnson & Cowan, 1974). (The “INTRODUCTION” contains details on House Sparrow introductions.) European Starlings and House Sparrows are declining in Metro Vancouver and across North America for unknown reasons (Berigan et al., 2020; Fink et al., 2023). In North America and in Vancouver, people have strong positive and negative relationships with both species (Belaire et al., 2015; Broadhead, 1971; Griffin, 2009; Karnicky, 2011). To attempt to mask the native status of each species, we omitted demonyms in species names (i.e., presented American robin as *robin*, and European starling as *starling*). These omissions match how these species are commonly referred to (https://avibase.bsc‐eoc.org/species.jsp?avibaseid=94A44032).

**FIGURE 1:**
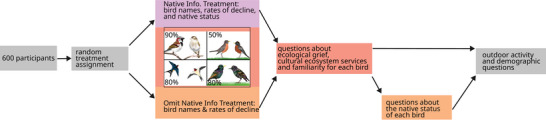
Experimental design of a survey of 600 residents of Metro Vancouver, British Columbia, Canada, about their perceptions of loss in relation to declines of native and non‐native bird species. The design included 2 treatments: native info. (participants were informed about the native status of each species), and omit native info. (participants were not informed about native status, but were asked about the native sptatus of each bird). Percentages, percent decline of that species over the last 25 years as reported to participants. Full survey in Appendix S3.

The first section of the survey contained 2 experimental treatments: the native information treatment stated the native status of each species, whereas the omit native information treatment did not state the status of the species but included a question about participants’ knowledge about the native status of each species (Figure 1). This design enabled us to make inferences about the concept of nativeness per se rather than just about native versus non‐native species. After a brief introduction (which either did or did not include information about native status), the first section included 4 sets of 14 questions (one set for each species). Each species‐specific 14‐question set included four 5‐level Likert‐scale questions about ecological grief (modified from Ágoston et al. [2022]), nine 5‐level Likert‐scale questions about losses of cultural ecosystem services (modified from Echeverri et al. [2020] and Gould et al. [2014]; see Table 1), and one 5‐po Likert‐scale question about familiarity with the species.

The second survey section included questions about tree declines and were unrelated to the current study (Eyster & Gould, 2025). The third section included questions about ecological knowledge, outdoor recreation activities, and demographics and a final free‐response question. Because formal ecological training and birdwatchers often tout the superiority of native species (Floyd, 2010a; Ricklefs & Relyea, 2018), we hypothesized that nativeness is more important to people who are ecology experts (including those with greater knowledge of ecology and those who regularly go birdwatching). In contrast, we hypothesized that nativeness matters less to people who set up feeders for wild birds because they may have a more amateur and aesthetic relationship with wildlife and simply have a desire to help birds survive (Jones, 2011). Similarly, we hypothesized that nativeness matters less to people who live in dense urban areas because they may have closer relationships with the non‐native species, which are often more abundant in core urban areas (Eyster, Chan, et al., 2024; Kowarik, 2011). So that we could more fully explain our results, we measured demographic variables associated with power, equity, and social justice (age, gender, education, income, race) (Biermann & Mansfield, 2014; Wardell‐Johnson, 2011) and variables that may shape or reflect relationships with birds (including familiarity with birds, environmental volunteering, and outdoor activities of walking, running, and biking). Outdoor walking and running may be particularly important because they are the most widespread outdoor activities in Canada (Statistics Canada, 2023).

Throughout the survey, we used visual sliders (bars that respondents could slide to the intended response to represent Likert answers) because they increase accessibility and accuracy in surveys (Nesbitt et al., 2023). To ensure the survey was interpreted as intended, we conducted think‐aloud pilots with colleagues and people who are not conservation experts, including Metro Vancouver residents (*n* = 10). Our analysis plan and hypotheses were preregistered on AsPredicted (Simmons et al., 2021; #13987).

### Statistical analyses

We cleaned and preprocessed data in R 4.3.2 and then fitted Bayesian cumulative probit models because Likert data are ordinal and nonmetric (Bürkner & Vuorre, 2019), with the brms 2.20.4 (Bürkner, 2018) interface to cmdstan 2.31.0 (Carpenter et al., 2017). We assessed model fit with $\hat{R}$ and posterior retrodictive checks. We adopted a Bayesian modeling framework because it allows more intuitive uncertainty interpretation, higher identifiability of complex models, and clearer information about parameters (Bürkner & Vuorre, 2019).

To characterize perceptions of non‐nativeness across both treatments, we created an index of non‐native information and beliefs. High values of this index indicated that either the species was non‐native and the participant received information that the species was non‐native (native information treatment [Figure 1]) or the participant stated they believed the species was non‐native (omit native information treatment [Figure 1]). Low values of the index indicated that either the species was native and the participant had received information that the species was native (native information treatment [Figure 1]) or the participant had stated they believed the species was native (omit native information treatment [Figure 1]). A neutral index value indicated that a participant in the omit treatment stated they did not know the nativeness status.

We built 2 models. The first was designed to test our preregistered hypotheses and the second to test exploratory hypotheses. The first model included the following input variables: familiarity with the species; species and its interaction with familiarity; whether the respondent feeds birds; frequency of birdwatching; non‐native belief and information index and its interactions with ecological knowledge; and respondent's residence urbanness (from very rural to high‐density urban).

Our second model was an exploratory analysis in which we attempted to determine what moderates the effects of native belief and information on feelings of ecological grief and cultural ecosystem service decline. To avoid multicollinearity (McElreath, 2020), we conducted Pearson and polychoric correlation analyses (Revelle, 2020) of all variables except those that had no discernible effect in the first model. Based on this correlation analysis, we included variables in the second model that had *r* ≤ 0.3 with all other input variables. If a correlation between 2 variables exceeded this value, we included only the variable that correlated more highly with the response variable. This model included the following input variables: familiarity with the species; species identity and its interactions with familiarity; birdwatching frequency; gardening frequency; sidewalk running or walking frequency; education; racial background; income; residence urbanness; gender; and non‐native belief and information index and its interactions with each of familiarity, gardening frequency, sidewalk running or walking frequency, education, racial background, income, and gender.

## RESULTS

### Sample details

Participant age, gender identity, residence urbanness, income, racial background, education, outdoor recreation activities, and ecological knowledge varied (Table 2).

**TABLE 2:** Sample frequencies of demographic, knowledge, and activity variables from a survey of 600 residents of Metro Vancouver, British Columbia, Canada, about their relationships with local declining native and non‐native birds.

Variable	Sample frequency
Gender identity	Women (53%), men (47%), nonbinary (*<*1%)
Age (years)	18–20 (3%), 21–30 (20%), 31–40 (21%), 41–50 (16%), 51–60 (16%), 61–70 (13%), 71–80 (10%), >81 (2%)
Residence urbanness	Very rural (3%), rural and exurban (5%), suburban or low‐density urban (30%), medium density urban (31%), high‐density urban (32%)
Income (CAD)	*<*$25,000 (10%), $25,000–$49,999 (16%), $50,000–$99,999 (36%), $100,000–$199,999 (32%), *>*$200,000 (6%)
Race	Black (3%), East Asian (19%), First Nations, Inuit, or Metis (2%), Latin American (1%), Middle Eastern (2%), multiple or other (6%), prefer not to say (4%), South Asian (7%), Southeast Asian (3%), White (55%)
Education	Some primary (*<*1%), completed primary (*<*1%), some secondary (3%), completed secondary (15%), vocational or some university (28%), university bachelor's degree (37%), graduate or professional degree (16%)
Bird feeding	Yes (29%), no (71%)
Birdwatching frequency	Never (56%), 1–10 times per year (24%), 10–30 times per year (9%), weekly (6%), daily or several times a week (6%)
Gardening	Never (38%), 1–10 times per year (23%), 10–30 times per year (10%), weekly (16%), daily or several times a week (14%)
Environmental volunteering or stewardship frequency	Never (75%), 1–10 times per year (15%), 10–30 times per year (5%), weekly (4%), daily or several times a week (1%)
Sidewalk running or walking frequency	Never (10%), 1–10 times per year (16%), 10–30 times per year (11%), weekly (24%), daily or several times a week (40%)
Trail running, walking, hiking, or snowshoeing on trails frequency	Never (22%), 1–10 times per year (30%), 10–30 times per year (17%), weekly (17%), daily or several times a week (14%)
Road bicycling frequency	Never (57%), 1–10 times per year (22%), 10–30 times per year (10%), weekly (7%), daily or several times a week (5%)
Mountain or gravel biking frequency	Never (73%), 1–10 times per year (15%), 10–30 times per year (6%), weekly (5%), daily or several times a week (1%)
Ecological knowledge	Very low (12%), low (30%), medium (46%), high (10%), very high (2%)

*Note*: Percentages may not add up to 100 due to rounding.

### Preregistered methods

**FIGURE 2:**
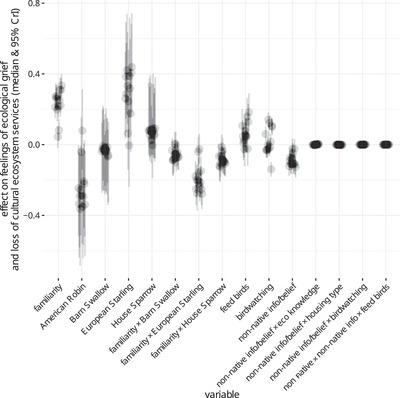
Results of a preregistered analyses showing the effects of the hypothesized variables on feelings of loss based on data from a survey of 600 residents of Metro Vancouver, British Columbia, Canada. For each variable or interaction, the 13 medians and credible intervals (clumps of gray dots and bars, respectively) show that variable's effects on the 13 dimensions of ecological grief and cultural ecosystem service loss (detailed results in Appendix S1). The more positive the effect, the stronger the feelings of grief or loss.

Overall, responses varied across the ecological grief and cultural ecosystem service questions (Figure 2). As hypothesized, decreases in birds that were more familiar evoked greater feelings of loss (Figure 2). When accounting for the effects of familiarity with each species, European starlings evoked greatest loss, then house sparrows, barn swallows, and, lastly, American robins (Figure 2). People who were more familiar with American robins felt more loss for this species (reference level for familiarity in Figure 2), but people who were more familiar with barn swallows, European starlings, and house sparrows showed mixed or less loss for these species (Figure 2).

As hypothesized, feeding birds and birdwatching both had significant effects. Feeding birds and frequency of birdwatching had mostly positive and mixed effects on feelings of loss, although birdwatchers felt less decrease in joy associated with bird declines (Figure 2, Appendix S1).

As hypothesized, information or beliefs that a species was non‐native had negative effects on some feelings of loss, including identity, joy, and connection with memories (Appendix S1). Contrary to our hypotheses, this effect of non‐native species information and beliefs was not moderated by ecological knowledge, residence urbanness, frequency of birdwatching, or bird feeding (Figure 2).

### Exploratory analyses

Our exploratory analysis showed that running/waking on sidewalks had a significant effect on feelings of loss, but in different ways than birdwatching (Appendix S2). For example, birdwatching—but not sidewalk running/walking—had a large effect on feelings of spiritual disruption (Appendix S2). In contrast, sidewalk running/walking—but not birdwatching—had a large effect on a sense of loss of neighborhood beauty (Appendix S2). Gardening, gender, and income did not have significant main effects, but education, residence urbanness, and race did for particular dimensions of loss (Appendix S2).

**FIGURE 3:**
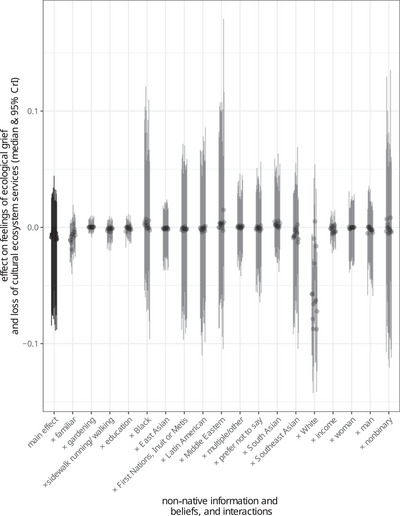
Selected results of exploratory analysis showing the effects of information or belief that a species was non‐native (main effect) and the interaction of this variable with other demographic and activity variables on feelings of ecological grief and loss of cultural ecosystem services based on a survey of 600 residents of Metro Vancouver, Canada, about feelings of loss in relation to native and non‐native species declines. Each of the 13 medians and 95% credible intervals (clumped gray dots and bars, respectively) represent the effects associated with each of the 13 ecological grief and cultural ecosystem service loss questions. Full model results are shown in Appendix S2. The more positive the effect, the stronger the feelings of grief or loss.

We found no main effect of non‐native belief and information on feelings of loss after accounting for its interactions with familiarity, gardening, sidewalk running/walking, education, race, income, and gender (Figure 3). However, White people reported fewer feelings of loss for declines in species they knew were non‐native—that is, 95% credible intervals for parameters representing the interaction between White and non‐native belief and information were <0 for 9 of 13 dimensions of loss (Figure 3). These 9 dimensions included those related to ecological equilibrium, sense of home, neighborhood excitement, neighborhood beauty, impacts to other wildlife, care for generations, fright, sense of loss, and sadness (Appendix S2). Of these, the effects for neighborhood beauty and sense of loss were particularly strong (Appendix S2). The interactions of non‐native belief and information with familiarity had neutral and marginally negative effects, and its interactions with each of income, gender, and education had mostly neutral to very weak and uncertain negative effects (Figure 3).

## DISCUSSION

Many non‐native bird species in Metro Vancouver, Canada, have declined precipitously, but their non‐native status did not diminish the feelings of loss experienced by local non‐White residents. In contrast, White respondents reported diminished feelings of loss about declines of birds they thought were non‐native (Figure 3). Not only may the nativeness concept not accurately describe ecological relationships (Chew & Hamilton, 2011; Davis et al., 2011), but our results also suggest that treating nativeness as universally preferred or valued may not capture how many local residents think about and interact with nature.

Some might interpret the observed unimportance of nativeness as reflecting insufficient ecological understanding (i.e., that overall, White people had greater ecological understanding to know that native species are ecologically better than non‐native species). Yet, we accounted for education, income, and ecological knowledge in our models, and these had null to marginal interactions with nativeness information and knowledge. Moreover, our experimental design allowed us to ensure that our results were not due to idiosyncratic and particular relationships with a species but were instead due to the perceived *native* status of that species. This experimental design also enabled us to measure the effects of native status implicitly, which, given that most people may not regularly think about the nativeness of animals, may yield more informative results (Schultz & Tabanico, 2007).

Our study differed from other studies because we separated perceptions of non‐native species by socioeconomic group, particularly relative to marginalization (Kapitza et al., 2019; c.f. Fischer et al., 2014; Straka et al., 2022), as recommended by Wardell‐Johnson (2011). Kapitza et al. (2019) reviewed social perceptions of invasive species and found scant coverage of perspectives of marginalized groups. This focus on the general population may be why so many studies show widely held negative perceptions of non‐native species and why our first model (which did not account for differences between privileged and marginalized groups) supported this common result (Bhattacharyya & Larson, 2014).

Our study joins a small but growing body of research that emphasizes the importance of investigating the diverse relationships that people have with non‐native species (Ravi & Hiremath, 2024). This research demonstrates that perceptions of non‐native species can be tightly tied to settler‐colonial and anti‐human‐immigrant beliefs (Coates, 2006) (consistent with the concept's British history [Chew & Hamilton, 2011]) and suggests that classed, gendered, racialized, and colonial power relations may thus shape perceptions of non‐native species (Bhattacharyya & Larson, 2014; Carruthers et al., 2011; Robbins, 2004). For example, results of a study in Zimbabwe showed that an expanding non‐native species may help empower female farmers (Kachena & Shackleton, 2024). Similarly, a study in British Columbia documented how Tsilhqot'in First Nation members treat non‐native free‐roaming horses as integral to their livelihoods and cultures. The First Nation members also recognize similarities between the prejudicial attitudes that settler governments have for both First Nations and free‐roaming and wild horses (Bhattacharyya & Larson, 2014). Conflicts over free‐roaming and wild horses are not just about the horses: “The link between free‐roaming horses and political control of the land dates back as far as the history of settlement by Euro‐Canadians and the establishment of cattle ranching as a major part of the Chilcotin economy” (Bhattacharyya & Larson, 2014, p. 674).

**FIGURE 4:**
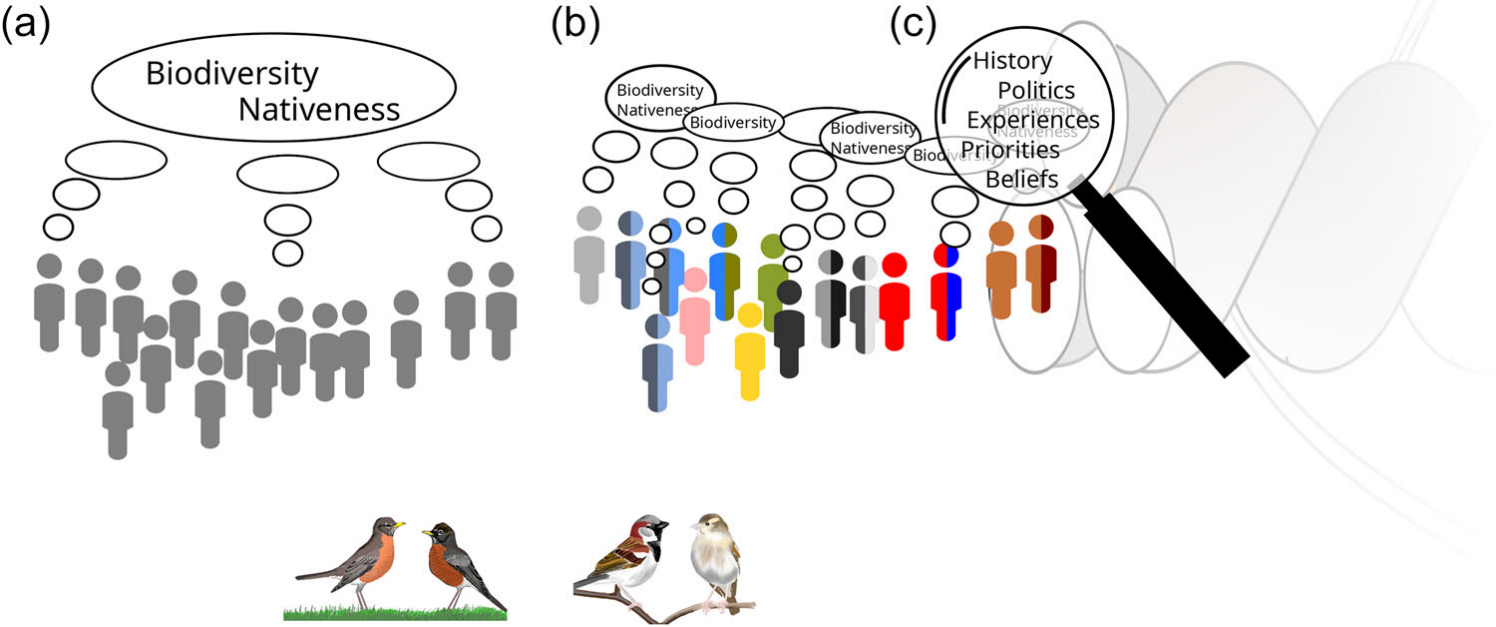
How results of a survey of 600 residents of Metro Vancouver, British Columbia, Canada, on their perceptions of loss in relation to declines of native and non‐native bird species inform conservation. (a) Conservationists thinking about species like House Sparrows and American Robins (bottom) and making the common assumption that all people define and think about concepts like biodiversity and nativeness uniformly because those concepts are objective and neutral. (b) Instead, nativeness and biodiversity are not objective concepts; they have histories and associated with politics. Native species are perceived based on these factors, and the desire to conserve them may vary according to identity and structures of marginalization. Because how one uses these concepts affects ecological and human communities, unquestioned reliance on scientists’ understandings of widely used concepts in conservation may have unintended consequences. (c) These unintended consequences might be avoided if conservationists carefully examine conservation concepts in light of their histories and definitions, and survey people who may be affected by conservation actions.

Our quantitative results complement and converge with a larger critical social science literature on nativeness. Our results’ suggestion that *nativeness* is not a neutral, purely ecological concept, but one entangled in identity and society (Figure 4), resonates with critical scholarship from diverse disciplines. One geographer wrote about plant nativeness: “[T]he boundary ostensibly being drawn around plants in Australia is in fact the boundary around humans, between humans and the rest of nature… it is even a boundary around the subset of humans who happen to be British colonisers and trained botanists. When analysed closely, characterisations such as nativeness tell us more about human bounding practices than anything inherent about the plants and their evolutionary processes” (Head, 2012, p. 171). According to an anthropologist, “The short answer to how scientists think about ‘natives’ is now: within parameters… of time, agency, and the politics of nature and culture in particular places” (Helmreich, 2005, p. 125). Similarly, and consistent with our argument that *native* and *non‐native* categories may have unintended racialized meanings and consequences, anthropologists Martin and Trigger (2015, p. 290) conclude that, “our research seeks to open up options for more creative adaptations to changing environments into the future, focusing on particular qualities of species rather than their categorizations as ‘introduced’ or ‘native’, always acknowledging the inclusion of humans within nature rather than apart from it.” Finally, interdisciplinary scholar Subramaniam (2001, p. 34) underscores how the whole idea of *native* is steeped in human marginalization and arguments over identity: “The battle against exotic and alien plants is a symptom of a campaign that misplaces and displaces anxieties about economic, social, political, and cultural changes onto outsiders and foreigners.”

This enmeshing of non‐native species in local communities, whether horses in Tsilhqot'in First Nation or European starlings in Metro Vancouver, is consistent with the emerging idea of novel, recombinant, or hybrid ecologies (Rotherham, 2017). “Many human aspirations for nature conservation and our subjective perceptions of what is ‘good’ are based on the ideas of stability and continuity. However, it is increasingly clear that whether or not we like it, the world is not stable and indeed, is getting less so” (Rotherham, 2017, p. xii; see also Floyd, 2010b). Ecologies are changing, and conservationists ought to better understand the consequences of these changes.

A strength of our study was our use of real ecological changes in our surveys, rather than hypothetical scenarios (Dubois & Fraser, 2013; Straka et al., 2022; Table 1). This ensured that our questions had ecological validity and could pique and validate people's experiences with bird declines. For example, one participant wrote in the optional free response question at the end of the survey, “It saddens me to see less if [sic] these birds as a child they were plentiful, now I get excited when I see robins in the yard it truly is sad.” Another wrote, “It is sad to see the bir[d] population is declining. The sparrow used to come to my home every day in bulk before around 8 years but now I can see them hardly.”

Moreover, by including ecologically documented population declines, our study could double not just as an academic exercise but also as an opportunity to translate ornithological research to the local community and ignite contemplation of local environmental issues. For example, one participant wrote, “thank you for the statistics although very negative.” Another wrote, “Thanks for this survey. It is sad to see such declines. Hopefully this survey will bring attention to how we treat our habitant [sic] wildlife.” And another wrote, “No thoughts just sadness.” By combining ecological data with questions about ecological grief and losses in ecosystem services, we were able to foster and characterize deep relationships with and feelings (Cunsolo, 2017a) about birds. Similar approaches might be fruitful for measuring other human–nature relationships (Eyster & Gould, 2025).

Our decision to treat each ecological grief and cultural ecosystem service item as an independent random effect (following ecological psychology and relational scholarship) rather than a constituent of a generalized index or construct enabled key insights. For example, this approach exposed the disperate ways that running or waking on sidewalks versus birdwatching affected relationships with declining bird species, with the former associated with aesthetic relationships and the latter associated with spiritual relationships (Appendix S2). Moreover, our decision to include sidewalk walking and running in our survey helped to ensure that we captured the main ways people interact with nature in our urban study area. Although conservation studies may often focus on traditional activities like hunting, angling, and birdwatching, walking is the most common outdoor activity in Canada, British Columbia, and the City of Vancouver. In Vancouver, 96% of people who recreate report walking (Statistics Canada, 2023), and walking and running were the most frequently practiced outdoor activities among our sample (Table 2). This broader conception of outdoor activities may improve understandings of relationships with nature, show how these relationships might be supported, and improve the well‐being of outdoor participants (Hicks et al., 2016; Martin et al., 2020; Outdoor Foundation, 2017; though focusing on walking can be ableist, see discussion in Taylor [2008]).

Amid large‐scale declines in non‐native species, our results raise important concerns about the equity of a nativeness‐based conservation ethic (Figure 4). The North American model of conservation has long elevated the ideas of powerful people; in some cases, the model aided colonization (Brune, 2020; Kashwan et al., 2021; Taylor, 2009, 2016). Our results suggest that the nativeness concept may continue to unintentionally enact landscapes that preferentially serve White people (Bhattacharyya & Larson, 2014), advance socioecological injustice (Yaka, 2019), and lead to green gentrification (Eyster, González, et al., 2024). Moreover, the week interaction effects between non‐native information and belief and income hint that conservation's elevation of native species may intersect with multiple forms of privilege. Our results are from a relatively small sample in one place, and further research in other places will likely provide fruitful insights.

Our results emphasize the urgent need for additional research that includes race, ethnicity, other markers of marginalization, and recreational experiences as key moderators of perceptions of and values associated with nativeness and other core conservation concepts. These perceptions and values might also be included in local decision‐making processes through so‐called environmental democracy, “defined as a participatory and ecologically rational form of collective decision‐making… …there is a very pressing *practical* justification for bringing environmentalism and democracy together in political terms; …we should want to make any expression of environmentalist values accountable to principles of human justice” (Mason, 2012, p. 2).

We hope our results suggest research directions that may have more immediate, on‐the‐ground impacts. Municipalities and other organizations might leverage the reciprocity associated with public surveys to simultaneously share ecological information with community members and, in return, receive information regarding public values and knowledge associated with nature. This could enable conservationists to make their work more widely known and better understand the likely relationship‐specific outcomes for nature and people of their conservation actions, priorities, and plans. Moreover, our findings might direclty inform environmental management policies of municipalities in Metro Vancouver.

More broadly, this work highlights how people and nature are inextricably intertwined (Packard, 1988). Landscapes and species are not as *natural* and separate from people as often assumed, as our discussion of Western Cattle‐Egrets demonstrates. Human‐aided transportation of a species from one place to another is not an exception to people–nature separation; rather, it is one of many links between people and nature. Recognition of these entanglements reveals opportunities forrelational research in ecological and social sciences. For instance, research that embraces and examines the inextricability of human and natural systems can question the assumption that one socially defined concept, such as nativeness, could ever be a panacea for guiding conservation (Figure 4).

## Supporting information



Supplementary Materials

## Data Availability

Anonymized raw data and custom code are archived on the Open Science Foundation platform at https://doi.org/10.17605/OSF.IO/W6VNQ.
