# Supplementary material for: How much biotic nativeness matters across human demographic groups
Source: Conserv Biol. 2025 Dec 27;40(2):e70197. doi: 10.1111/cobi.70197 (PMC13036309; doi:10.1111/cobi.70197)
Supplement: Supplementary file 1 — Supplementary Materials [file COBI-40-e70197-s001.pdf]

Supplementary information for:
How much biotic nativeness matters across human demographic groups

Published in *Conservation Biology* in 2025 by Harold N. Eyster & Rachelle K. Gould.
DOI: 10.1111/cobi.70197

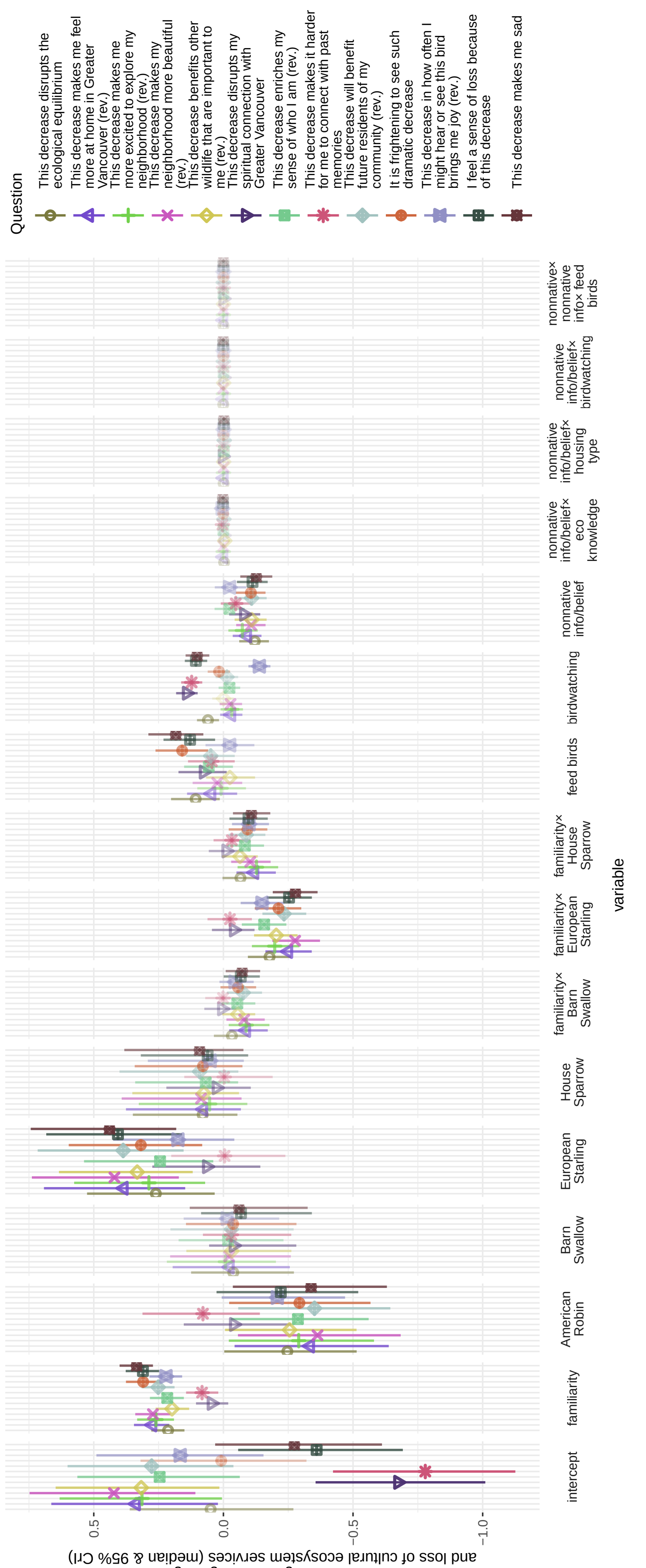

Figure Appendix S1: Full model results of pre-registered analysis of feelings of ecological grief and loss of cultural ecosystem services.

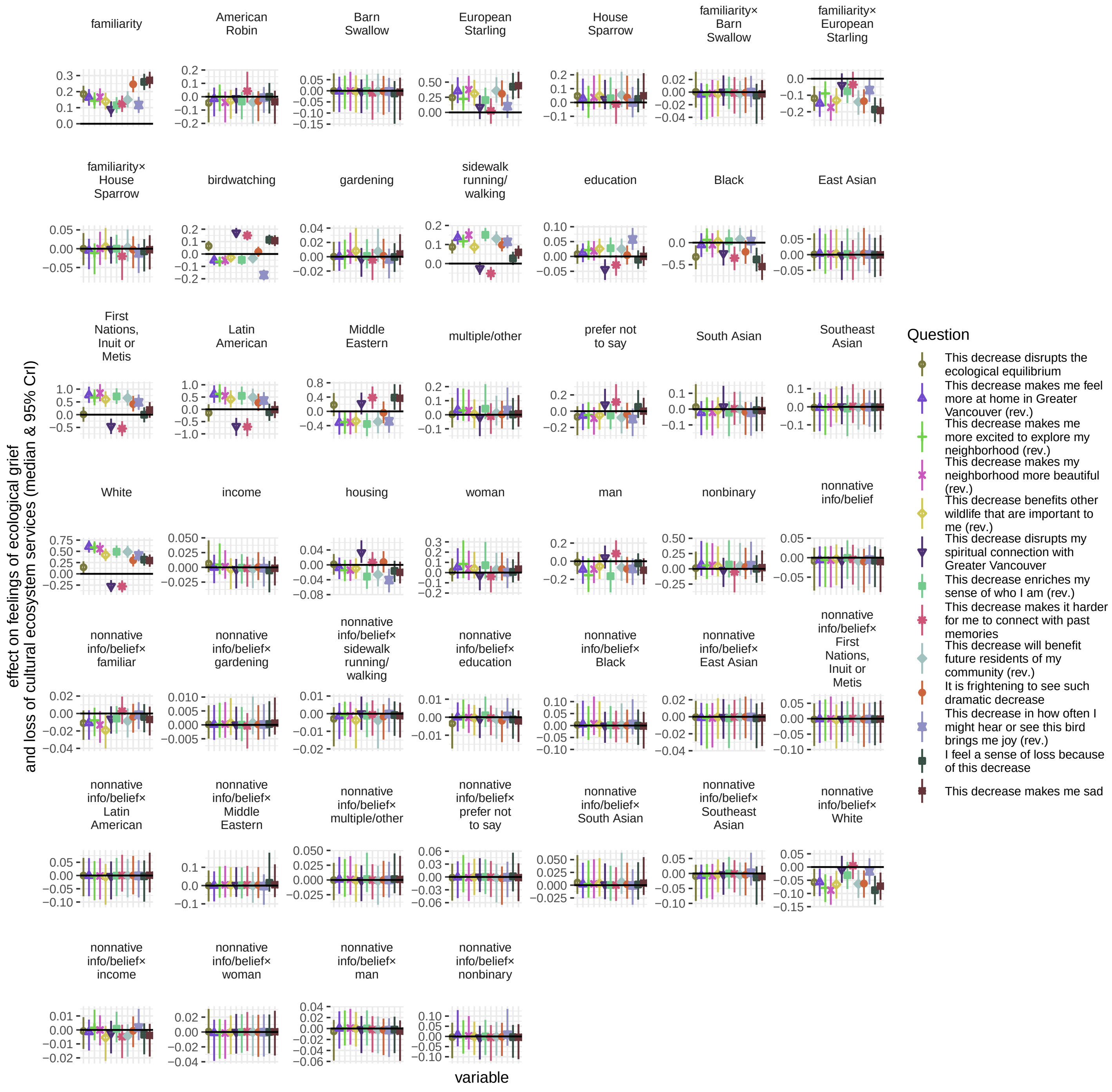

Figure Appendix S2: Full model results of exploratory analysis showing medians and 95% credible intervals (CrI) for effects of each variable on feelings of ecological grief and loss of cultural ecosystem services. Note the variable y-axis scales.

Appendix S3

[redacted]

consent

Title of Study: Meanings of wildlife trends
Principal Investigator (PI): [redacted]
Faculty Sponsor [redacted]
Funder: [redacted]
Introduction You are being invited to take part in this research study because you are a resident of Metro Vancouver, BC. This study is being conducted by [redacted]
Purpose: This research study aims to understand how people think about changes in urban wildlife in British Columbia.
Study Procedures: If you take part in the study, you will be shown a survey and asked to answer a series of questions about your experiences with birds and trees in Metro Vancouver. The survey should take approximately 10 minutes, and you will be asked to complete all questions. This survey requires only one session.
Benefits: As a participant in this research study, there may not be direct benefit to you; however, information from this study may benefit other people now or in the future.
Risks: We will not collect any information that will identify you to protect your confidentiality.
Costs: There will be no costs to you for participation in this research study.
Compensation: For taking part in this research study, you will be reimbursed for your time and inconvenience.
Confidentiality: All information collected about you during the course of this study will be stored without any identifiers. No one will be able to match you to your answers.
Voluntary Participation/Withdrawal: Taking part in this study is voluntary. You are free to not answer any questions or withdraw at any time. You may choose not to take part in this study, or if you decide to take part, you can change your mind later and withdraw from the study.
Questions: If you have any questions about this study now or in the future, you may contact heyster@uvm.edu If you have questions or concerns about your rights as a research participant, [redacted] It is recommended you print this information sheet for your records before continuing.

residence

Where do you currently live?

- ○ Vancouver, BC
- ○ Greater Vancouver/Metro Vancouver, BC
- ○ Somewhere else in British Columbia
- ○ I do not currently live in British Columbia

neutral

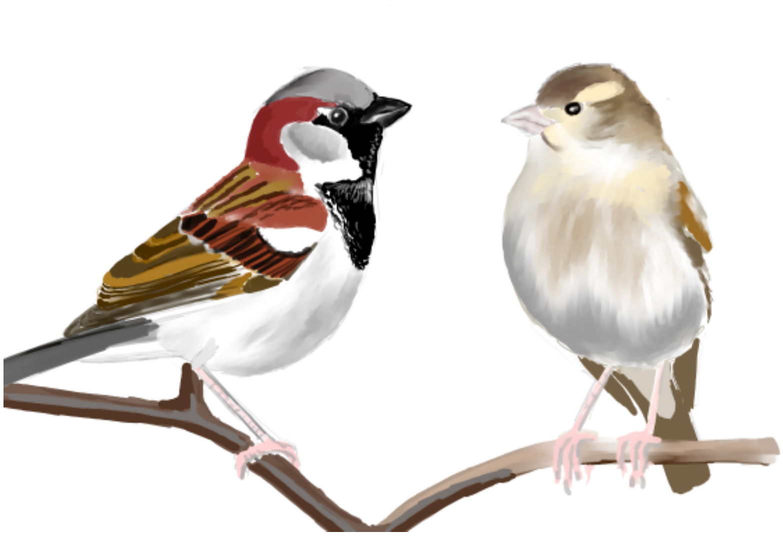

These birds are House Sparrows (sometimes just called "sparrows").

During the last 25 years, this species has decreased by 90% in Metro Vancouver. The following questions are about this bird and its change in population.

	Strongly disagree	Neutral/ don't know	Strongly agree
This decrease disrupts the ecological equilibrium			
This decrease makes me feel more at home in Greater Vancouver			
This decrease makes me more excited to explore my neighborhood			
This decrease makes my neighborhood more beautiful			
This decrease benefits other wildlife that are important to me			
This decrease disrupts my spiritual connection with Greater Vancouver			
This decrease enriches my sense of who I am			
This decrease makes it harder for me to connect with past memories			
This decrease will benefit future residents of my community			
It is frightening			

to see such dramatic decrease

This decrease in how often I might hear or see this bird brings me joy

I feel a sense of loss because of this decrease

This decrease makes me sad

	Definitely no	Neutral/ don't know	Definitely yes
I am familiar with this bird			

Please select 'strongly agree' to show you are paying attention to this question

- strongly disagree
- Somewhat disagree
- Neither agree nor disagree
- Somewhat agree
- Strongly agree

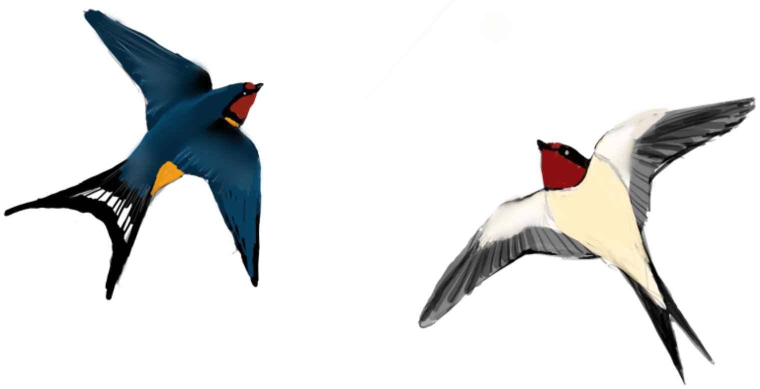

These birds are Barn Swallows.

During the last 25 years, this species has declined by 80% in Metro Vancouver. The following questions are about this bird and its change in population.

	Strongly disagree	Neutral/ don't know	Strongly agree
This decrease disrupts the ecological equilibrium			
This decrease makes me feel more at home in Greater Vancouver			
This decrease makes me more excited to explore my neighborhood			
This decrease makes my neighborhood more beautiful			
This decrease benefits other wildlife that are important to me			
This decrease disrupts my spiritual connection with Greater Vancouver			
This decrease enriches my sense of who I am			
This decrease makes it harder for me to connect with past memories			
This decrease will benefit future residents of my community			
It is frightening			

to see such dramatic decrease

This decrease in how often I might hear or see this bird brings me joy

I feel a sense of loss because of this decrease

This decrease makes me sad

	Definitely no	Neutral/ don't know	Definitely yes
I am familiar with this bird			

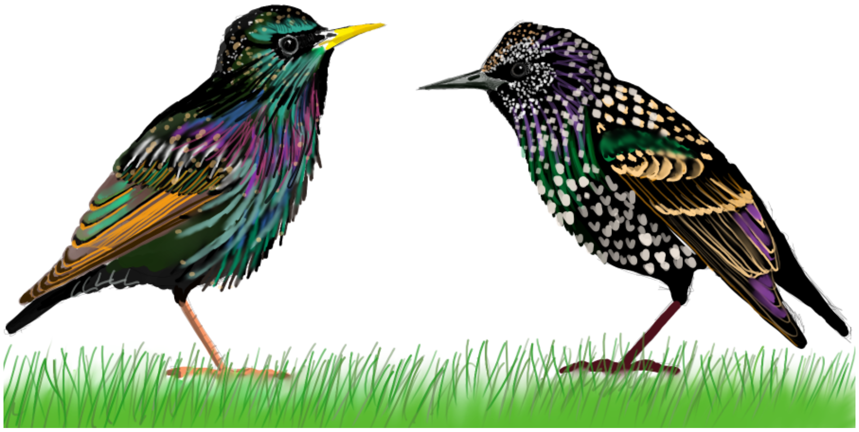

These birds are Starlings.

During the last 25 years, this species has declined by 80% in Metro Vancouver. The following questions are about this bird and its change in population.

	Strongly disagree	Neutral/ don't know	Strongly agree
This decrease disrupts the ecological equilibrium			
This decrease makes me feel more at home in Greater Vancouver			
This decrease makes me more excited to explore my neighborhood			
This decrease makes my neighborhood more beautiful			
This decrease benefits other wildlife that are important to me			
This decrease disrupts my spiritual connection with Greater Vancouver			
This decrease enriches my sense of who I am			
This decrease makes it harder for me to connect with past memories			
This decrease will benefit future residents of my community			
It is frightening			

to see such dramatic decrease

This decrease in how often I might hear or see this bird brings me joy

I feel a sense of loss because of this decrease

This decrease makes me sad

	Definitely no	Neutral/ don't know	Definitely yes
I am familiar with this bird			

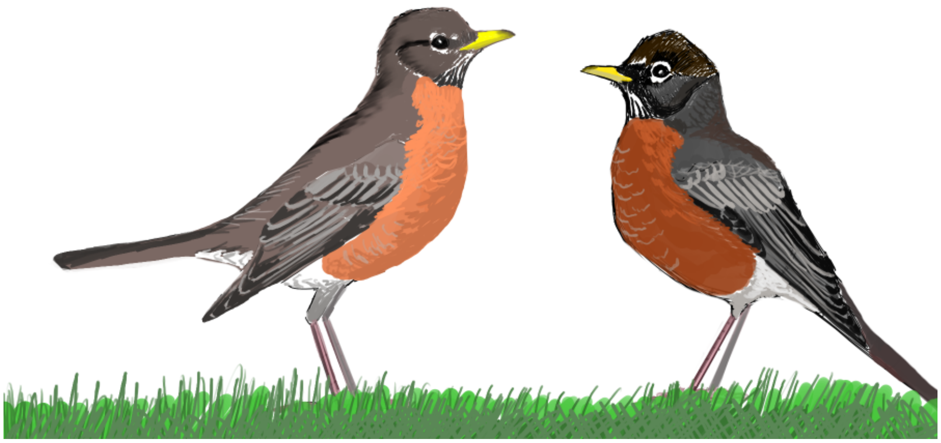

These birds are Robins.

During the last 25 years, this species has declined by 80% in Metro Vancouver. The following questions are about this bird and its change in population.

	Strongly disagree	Neutral/ don't know	Strongly agree
This decrease disrupts the ecological equilibrium			
This decrease makes me feel more at home in Greater Vancouver			
This decrease makes me more excited to explore my neighborhood			
This decrease makes my neighborhood more beautiful			
This decrease benefits other wildlife that are important to me			
This decrease disrupts my spiritual connection with Greater Vancouver			
This decrease enriches my sense of who I am			
This decrease makes it harder for me to connect with past memories			
This decrease will benefit future residents of my community			
It is frightening			

to see such dramatic decrease	
This decrease in how often I might hear or see this bird brings me joy	
I feel a sense of loss because of this decrease	
This decrease makes me sad	

	Definitely no	Neutral/ don't know	Definitely yes
I am familiar with this bird			

	Definitely no	Neutral/ don't know	Definitely yes
House Sparrows are native to North America			
Barn Swallows are native to North America			
Starlings are native to North America			
Robins are native to North America			

native

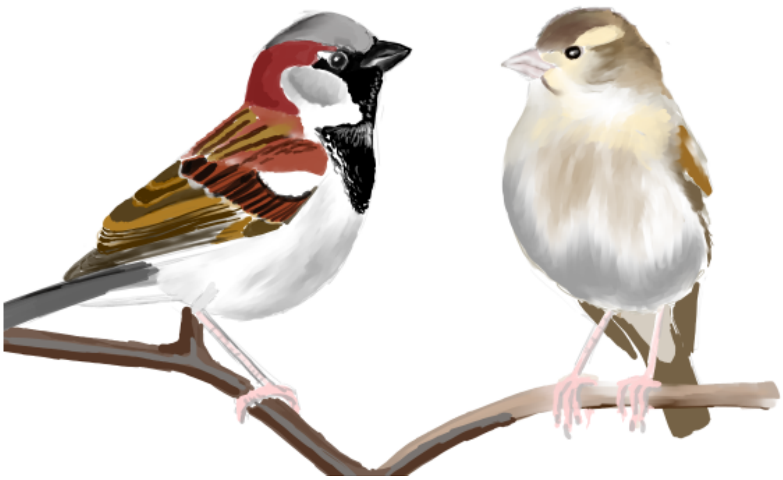

These birds are House Sparrows (sometimes just called "sparrows").

House Sparrows are not native to North America.

During the last 25 years, this species has decreased by 90% in Metro Vancouver. The following questions are about this bird and its change in population.

	Strongly disagree	Neutral/ don't know	Strongly agree
This decrease disrupts the ecological equilibrium			
This decrease makes me feel more at home in Greater Vancouver			
This decrease makes me more excited to explore my neighborhood			
This decrease makes my neighborhood more beautiful			
This decrease benefits other wildlife that are important to me			
This decrease disrupts my spiritual connection with Greater Vancouver			
This decrease enriches my sense of who I am			
This decrease makes it harder for me to connect with past memories			
This decrease will benefit future residents of my community			
It is frightening			

[illegible] to see such dramatic decrease

This decrease in how often I might hear or see this bird brings me joy

I feel a sense of loss because of this decrease

This decrease makes me sad

	Definitely no	Neutral/ don't know	Definitely yes
I am familiar with this bird			

Please select 'strongly agree' to show you are paying attention to this question

- strongly disagree
- Somewhat disagree
- Neither agree nor disagree
- Somewhat agree
- Strongly agree

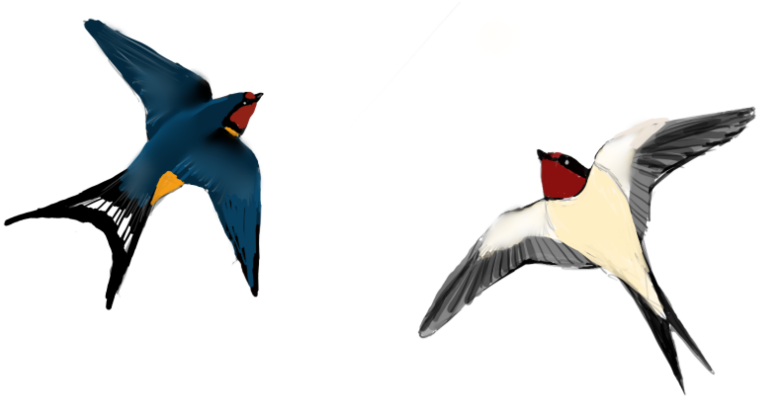

These birds are Barn Swallows.

Barn Swallows are native to North America.

During the last 25 years, this species has declined by 80% in Metro Vancouver. The following questions are about this bird and its change in population.

	Strongly disagree	Neutral/ don't know	Strongly agree
This decrease disrupts the ecological equilibrium			
This decrease makes me feel more at home in Greater Vancouver			
This decrease makes me more excited to explore my neighborhood			
This decrease makes my neighborhood more beautiful			
This decrease benefits other wildlife that are important to me			
This decrease disrupts my spiritual connection with Greater Vancouver			
This decrease enriches my sense of who I am			
This decrease makes it harder for me to connect with past memories			
This decrease will benefit future residents of my community			
It is frightening			

to see such dramatic decrease

This decrease in how often I might hear or see this bird brings me joy

I feel a sense of loss because of this decrease

This decrease makes me sad

	Definitely no	Neutral/ don't know	Definitely yes
I am familiar with this bird			

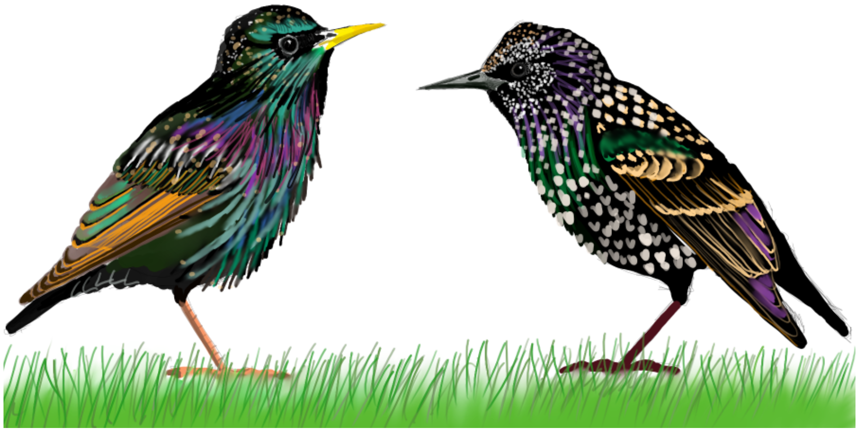

These birds are Starlings.

Starlings are not native to North America.

During the last 25 years, this species has declined by 80% in Metro Vancouver. The following questions are about this bird and its change in population.

	Strongly disagree	Neutral/ don't know	Strongly agree
This decrease disrupts the ecological equilibrium			
This decrease makes me feel more at home in Greater Vancouver			
This decrease makes me more excited to explore my neighborhood			
This decrease makes my neighborhood more beautiful			
This decrease benefits other wildlife that are important to me			
This decrease disrupts my spiritual connection with Greater Vancouver			
This decrease enriches my sense of who I am			
This decrease makes it harder for me to connect with past memories			
This decrease will benefit future residents of my community			
It is frightening			

to see such dramatic decrease

This decrease in how often I might hear or see this bird brings me joy

I feel a sense of loss because of this decrease

This decrease makes me sad

	Definitely no	Neutral/ don't know	Definitely yes
I am familiar with this bird			

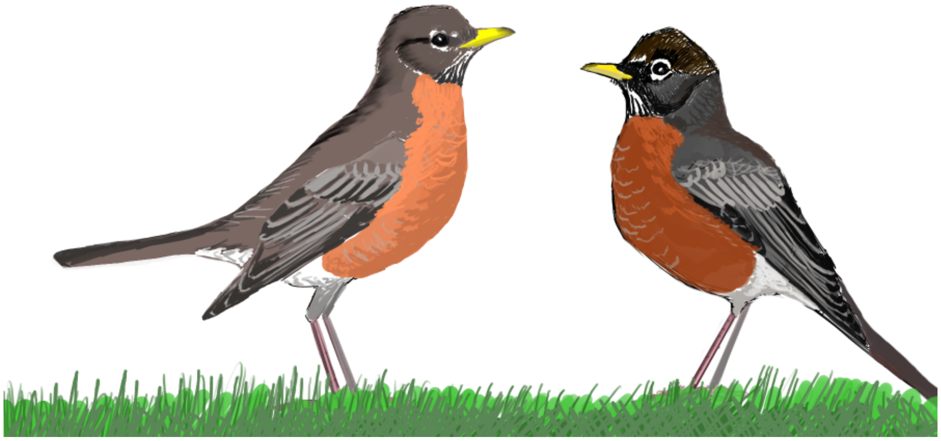

These birds are Robins.

Robins are native to North America.

During the last 25 years, this species has declined by 80% in Metro Vancouver. The following questions are about this bird and its change in population.

	Strongly disagree	Neutral/ don't know	Strongly agree
This decrease disrupts the ecological equilibrium			
This decrease makes me feel more at home in Greater Vancouver			
This decrease makes me more excited to explore my neighborhood			
This decrease makes my neighborhood more beautiful			
This decrease benefits other wildlife that are important to me			
This decrease disrupts my spiritual connection with Greater Vancouver			
This decrease enriches my sense of who I am			
This decrease makes it harder for me to connect with past memories			
This decrease will benefit future residents of my community			

It is frightening

to see such dramatic decrease

This decrease in how often I might hear or see this bird brings me joy

I feel a sense of loss because of this decrease

This decrease makes me sad

	Definitely no	Neutral/ don't know	Definitely yes
I am familiar with this bird			

tree

During the past 25 years, conifers—such as firs, cedars, pines, hemlocks, and spruces—have declined by 54% in Vancouver.

During the past 25 years, broad-leaf deciduous trees—such as cherries, maples, oaks, and alders—have declined by 9% in Vancouver.

The following questions are about these changes.

	Strongly disagree	Neutral/ don't know	Strongly agree
This decrease in conifers makes me sad			
This decrease in broadleaf deciduous trees makes me sad			

tree_swap

During the past 25 years, conifers—such as firs, cedars, pines, hemlocks, and spruces—have declined by 9% in Vancouver.

During the past 25 years, broad-leaf deciduous trees—such as cherries, maples, oaks, and alders—have declined by 54% in Vancouver.

The following questions are about these changes.

	Strongly disagree	Neutral/ don't know	Strongly agree
This decrease in conifers makes me sad			
This decrease in broadleaf deciduous trees makes me sad			

demographics

How would you rate your knowledge of ecology?

- very low
- low
- medium
- high
- very high

Do you feed wild birds at home?

- No
- Yes

During the last year, how often did you participate in the following activities?

	I don't do this activity	1-10x	10-30x	Weekly	Daily/several times a week
Walking, hiking, snowshoeing, or running, on trails	○	○	○	○	○
Bicycling on roads	○	○	○	○	○
Environmental volunteering or stewardship	○	○	○	○	○
Birdwatching or birding	○	○	○	○	○
Mountain biking or gravel biking	○	○	○	○	○
Gardening	○	○	○	○	○
Running or walking, on sidewalks	○	○	○	○	○

What is the highest level of education you have completed?

- ○ Some Primary
- ○ Completed Primary
- ○ Some Secondary
- ○ Completed Secondary
- ○ Vocational or some university
- ○ University Bachelors Degree
- ○ Graduate or Professional Degree (MA, MS, MBA, PhD, JD, MD, DDS etc.)

How do you identify? (You may select as many as apply to you.)

- ☐ Black
- ☐ First Nations, Inuit or Metis
- ☐ White
- ☐ Middle Eastern
- ☐ East Asian
- ☐ South Asian
- ☐ Latin American
- ☐ Southeast Asian
- ☐ Prefer not to say
- ☐ ______ Other (please specify):

What was your total household income before taxes during the past 12 months?

- ○ Less than $25,000
- ○ $25,000-$49,999
- ○ $50,000-$99,999
- ○ $100,000-$199,999
- ○ More than $200,000

What kind of environment do you currently live in?

- ○ Very rural
- ○ Rural / exurban
- ○ Suburban or low-density urban
- ○ medium-density urban
- ○ high-density urban

What is your age?

- 18-20 years old
- 21-30 years old
- 31-40 years old
- 41-50 years old
- 51-60 years old
- 61-70 years old
- 71-80 years old
- 81+ years old

What is your gender identity?

- Female
- Male
- Non-binary
- Other, or prefer not to say

Block 5

If there are any additional thoughts you would like to share, or feedback about your experience completing this survey, please use the space below to record them.

Powered by Qualtrics
